# In Vivo Sublayer Analysis of Human Retinal Inner Plexiform Layer Obtained by Visible-Light Optical Coherence Tomography

**DOI:** 10.1167/iovs.63.1.18

**Published:** 2022-01-13

**Authors:** Zeinab Ghassabi, Roman V. Kuranov, Joel S. Schuman, Ronald Zambrano, Mengfei Wu, Mengling Liu, Behnam Tayebi, Yuanbo Wang, Ian Rubinoff, Xiaorong Liu, Gadi Wollstein, Hao F. Zhang, Hiroshi Ishikawa

**Affiliations:** 1 Department of Ophthalmology, NYU Langone Health, NYU Grossman School of Medicine, New York, New York, United States; 2 Department of Biomedical Engineering, Northwestern University, Evanston, Illinois, United States; 3 Opticent Inc., Evanston, Illinois, United States; 4 Department of Biomedical Engineering, New York University Tandon School of Engineering, Brooklyn, New York, United States; 5 Neuroscience Institute, NYU Langone Health, NYU Grossman School of Medicine, New York, New York, United States; 6 Department of Biology, University of Virginia, Charlottesville, Virginia, United States; 7 Department of Ophthalmology, Casey Eye Institute, Oregon Health & Science University, Portland, Oregon, United States; 8 Department of Electrical and Computer Engineering, New York University Tandon School of Engineering, Brooklyn, New York, United States; 9 Center for Neural Science, NYU College of Arts and Sciences, New York, New York, United States; 10 Department of Physiology and Neuroscience, NYU Langone Health, NYU Grossman School of Medicine, New York, New York, United States

**Keywords:** vis-OCT, IPL sublayers, spectroscopy, OCT, inner plexiform layer

## Abstract

**Purpose:**

Growing evidence suggests that dendrite retraction or degeneration in a subpopulation of the retinal ganglion cells (RGCs) may precede detectable soma abnormalities and RGC death in glaucoma. Visualization of the lamellar structure of the inner plexiform layer (IPL) could advance clinical management and fundamental understanding of glaucoma. We investigated whether visible-light optical coherence tomography (vis-OCT) could detect the difference in the IPL sublayer thicknesses between small cohorts of healthy and glaucomatous subjects.

**Method:**

We imaged nine healthy and five glaucomatous subjects with vis-OCT. Four of the healthy subjects were scanned three times each in two separate visits, and five healthy and five glaucoma subjects were scanned three times during a single visit. IPL sublayers were manually segmented using averaged A-line profiles.

**Results:**

The mean ages of glaucoma and healthy subjects are 59.6 ± 13.4 and 45.4 ± 14.4 years (*P* = 0.02.) The visual field mean deviations (MDs) are −26.4 to −7.7 dB in glaucoma patients and −1.6 to 1.1 dB in healthy subjects (*P* = 0.002). Median coefficients of variation (CVs) of intrasession repeatability for the entire IPL and three sublayers are 3.1%, 5.6%, 6.9%, and 5.6% in healthy subjects and 1.8%, 6.0%, 7.7%, and 6.2% in glaucoma patients, respectively. The mean IPL thicknesses are 36.2 ± 1.5 µm in glaucomatous and 40.1 ± 1.7 µm in healthy eyes (*P* = 0.003).

**Conclusions:**

IPL sublayer analysis revealed that the middle sublayer could be responsible for the majority of IPL thinning in glaucoma. Vis-OCT quantified IPL sublayers with good repeatability in both glaucoma and healthy subjects.

Glaucoma is a neurodegenerative disease characterized by retinal ganglion cell (RGC) death and axon degeneration, leading to vision loss.^1^^,^^2^ Over the past two and a half decades, optical coherence tomography (OCT) provided repeatable in vivo quantitative thickness assessment of retinal nerve fiber layer (RNFL) and combined ganglion cell layer (GCL) and inner plexiform layer (IPL) referenced as GCIPL, or GCL, IPL, and macular RNFL referenced as ganglion cell complex (GCC), which are clinically useful biomarkers for glaucoma assessment.^3^^–^^7^

RGCs have complex yet characteristic dendritic morphology that determines how they receive and transmit visual information.^8^ Specifically, the inner neurons form synapses with RGC dendrites in the IPL, which can be divided into ON and OFF sublamellae, reflecting the functional segregation of the ON and OFF pathways.^8^^,^^9^ ON RGCs have dendritic arbors in the inner region of the IPL, whereas OFF RGC dendrites co-localize with OFF bipolar axonal terminals in sublamella *a*.^10^^,^^11^ ON-OFF RGCs have dendrites arborizing in both sublamellae *a* and *b* of the IPL and respond to both light onset and offset.^12^ Since IPL consists of various types of dendrites, a quantitative analysis of the IPL sublayer structure may provide additional information about glaucomatous insults to the retinal neural tissues in vivo.

However, the changes of the ON and OFF IPL sublamellae in glaucoma is controversial. It was shown in ex vivo studies in mice that the IPL layer can be the first location of the structural glaucomatous damage.^13^ Although some studies indicate that an OFF sublamella is affected in glaucoma,^14^^,^^15^ whereas other studies suggested that an ON sublamella was susceptible to the optic nerve crush (ONC).^16^^,^^17^

Recently, the speckle reduction technique in both the conventional near-infrared (NIR)^18^^,^^19^ and visible light spectra (vis-OCT)^20^^–^^23^ allowed delineating IPL sublayers. Specifically, three hyper-reflective and two hyporeflective bands in the IPL were revealed, corresponding well with the reported anatomical division of the IPL into five strata.^24^^,^^25^

In this work, among those five IPL bands, we used speckle-reduction vis-OCT to identify three IPL sublayers using the minimal signal intensity of the two hyporeflective bands and the outer IPL boundaries. We measured IPL sublayer thickness in a small cohort of glaucoma and healthy subjects to evaluate vis-OCT IPL sublayer imaging repeatability and potentially quantify dendritic degeneration of the RGCs in glaucoma.

## Methods

### Subjects Recruiting

The study was approved by the New York University Langone Health institutional review board and complied with the tenets of the Declaration of Helsinki. Informed consent was received from all subjects before imaging. Both men and women of all races/ethnicities ages 18 years or older were eligible for the study.

Fourteen eyes of 14 subjects (nine healthy and five glaucomatous) were imaged. Five healthy and five glaucoma subjects participated in the intrasession repeatability study; four healthy subjects participated in the intersession repeatability study. For the intrasession repeatability study, both glaucoma and healthy subjects were imaged three times in a single visit. For intersession repeatability, four healthy subjects were imaged three times each in two separate visits.

All subjects were tested with visual field (VF), commercial near-infrared (NIR)-OCT, and visible light (vis)-OCT. VFs were tested with the Swedish interactive thresholding algorithm 24-2 perimetry (SITA standard; Humphrey Field Analyzer; Zeiss, Dublin, CA, USA). Reliable VFs were considered tests with less than 33% fixation losses and false-positive and false-negative responses. Mean deviation (MD) was used for the analysis. All subjects were imaged with commercial NIR-OCT (Cirrus HD-OCT; Zeiss) using the optic nerve head (ONH) cube of 200 × 200 scans. Global mean circumpapillary RNFL thickness was used for analysis.

Inclusion criteria, common to both the healthy and glaucoma groups, consisted of reliable standard automated perimetry (SAP) defined as <20% fixation losses and <33% false-positive and false-negative errors, the spherical equivalent refractive error between −4.00 and +4.00 D sphere, best-corrected visual acuity of 20/40 or better, age ≥18 and ≤80 years, and no prior history of intraocular surgery.

Inclusion criteria for the healthy group were intraocular pressure (IOP) ≤ 21 mm Hg, normal appearance of the optic nerve head and RNFL, normal SAP defined as a glaucomatous hemifield test within normal limits, pattern SD (PSD) within 95% confidence interval limits, and cup-to-disc ratio difference < 0.2 in both vertical and horizontal dimensions.

Inclusion criteria for the glaucoma group consisted of glaucomatous optic neuropathy and corresponding abnormal VF defined as abnormal glaucomatous hemifield test and PSD outside 95% normal limits. The glaucoma group patients required the glaucomatous damage to be in the superior of OCT and the inferior hemifield. All patients were familiar with SAP testing from earlier exposure to at least two VF examinations.

Exclusion criteria for both groups were a history of intraocular/laser surgery, existing retinal pathologies, nonglaucomatous optic neuropathy, uveitis, ocular trauma, or diabetes. Participants with systemic hypertension were included unless they were diagnosed with hypertensive retinopathy. Participants with unreliable VF results and poor-quality spectral-domain OCT scans resulting from occluding medial opacities were excluded from this study.

### Vis-OCT Imaging

**Figure 1.**
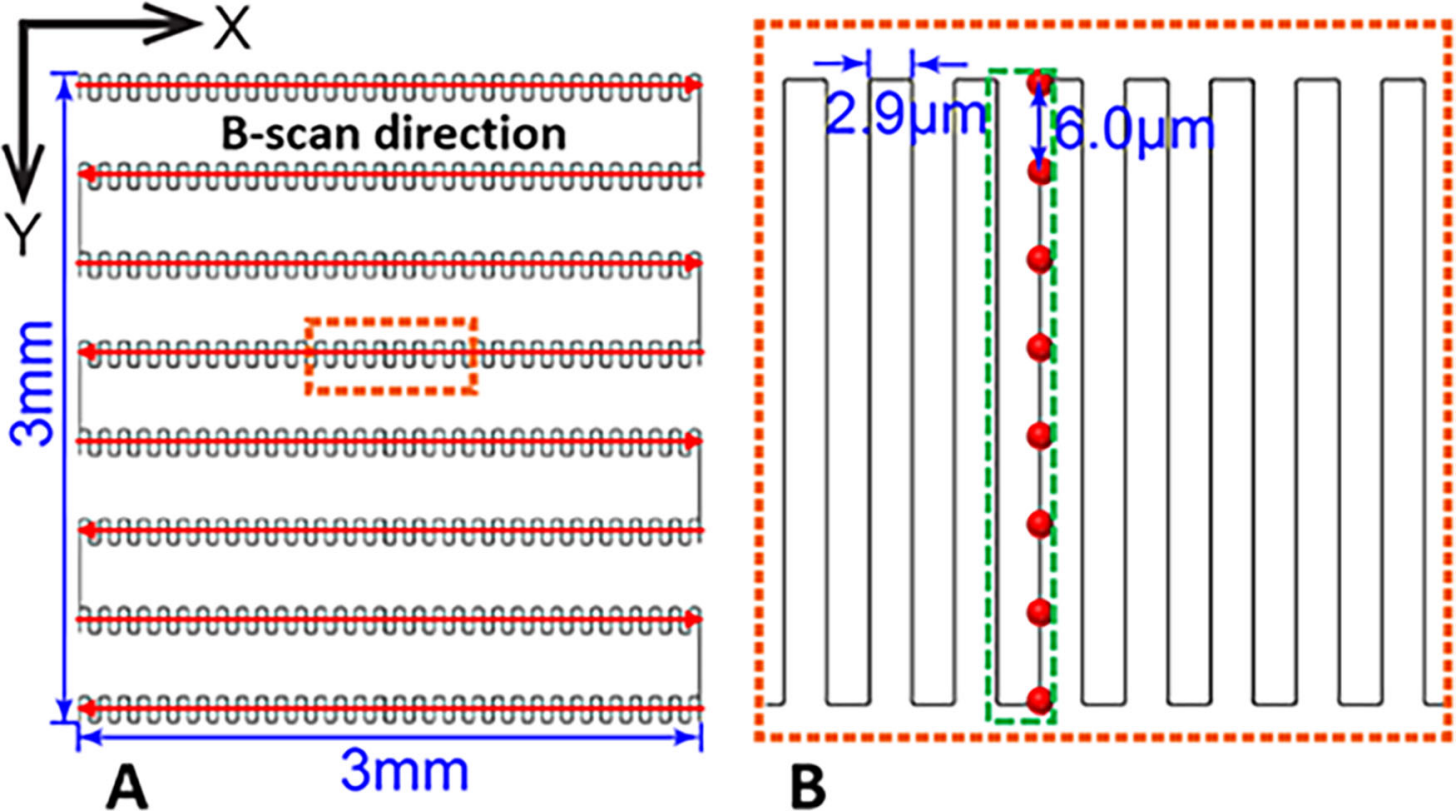
(**A**) Illustration of the overall speckle-reduction raster scanning protocol in vis-OCT. (**B**) Illustration of srA-line acquisition as highlighted by the *dashed box* in panel **A**. The *red dots* indicate the spatial locations of each regular A-line.

We used the Aurora X1 vis-OCT system (Opticent Inc., Evanston, IL)^22^ to image IPL sublayers. The system was running at a rate of 25,000 A-scans/sec. The incident power was set below 250 µW, which was within the laser safety limit defined by the ANSI standard^26^^,^^27^ for the scan pattern shown in Figure 1. Meanwhile, our vis-OCT has also been certified by the Food and Drug Administration as a nonsignificant risk device for laser safety. Vis-OCT irradiation power was measured using a calibrated power meter (PM100D; Thorlabs, Newton, NJ, USA) before each imaging session.

We used a unique speckle-reduction raster scanning protocol for vis-OCT image acquisition.^22^ The scanning covers a volume of 3 × 3 × 1.2 mm^3^ (horizontal × vertical × axial with 8192 × 8 × 1024 pixels, respectively) in the retina centered at the foveola along the x-axis (Fig. 1a). Along the y-axis, the data set was acquired from the superior part of the fovea, where one B-scan (the last or second to last B-scan) crossed the foveola. Along the x-axis, we acquired a speckle-reduced B-Scan (srB-scan) image by vertically translating the vis-OCT scanning beam along the y-axis, as shown in Figure 1b. Such a spatial translation consisted of eight uncorrelated A-lines, as highlighted by the red dots in Figure 1b, with an interval of 6 µm, and the spatial interval between two vertical translations was 2.9 µm, as shown in Figure 1b. For every two vertical translations, we calculated a speckle-reduced A-line (srA-line) by averaging 16 regular A-lines as highlighted by the green dashed box in Figure 1b. Therefore each srB-scan contains 512 srA-lines, and the spatial interval between the srB-scans is 375 µm.

### Post-Processing and Measurement Sampling

**Figure 2.**
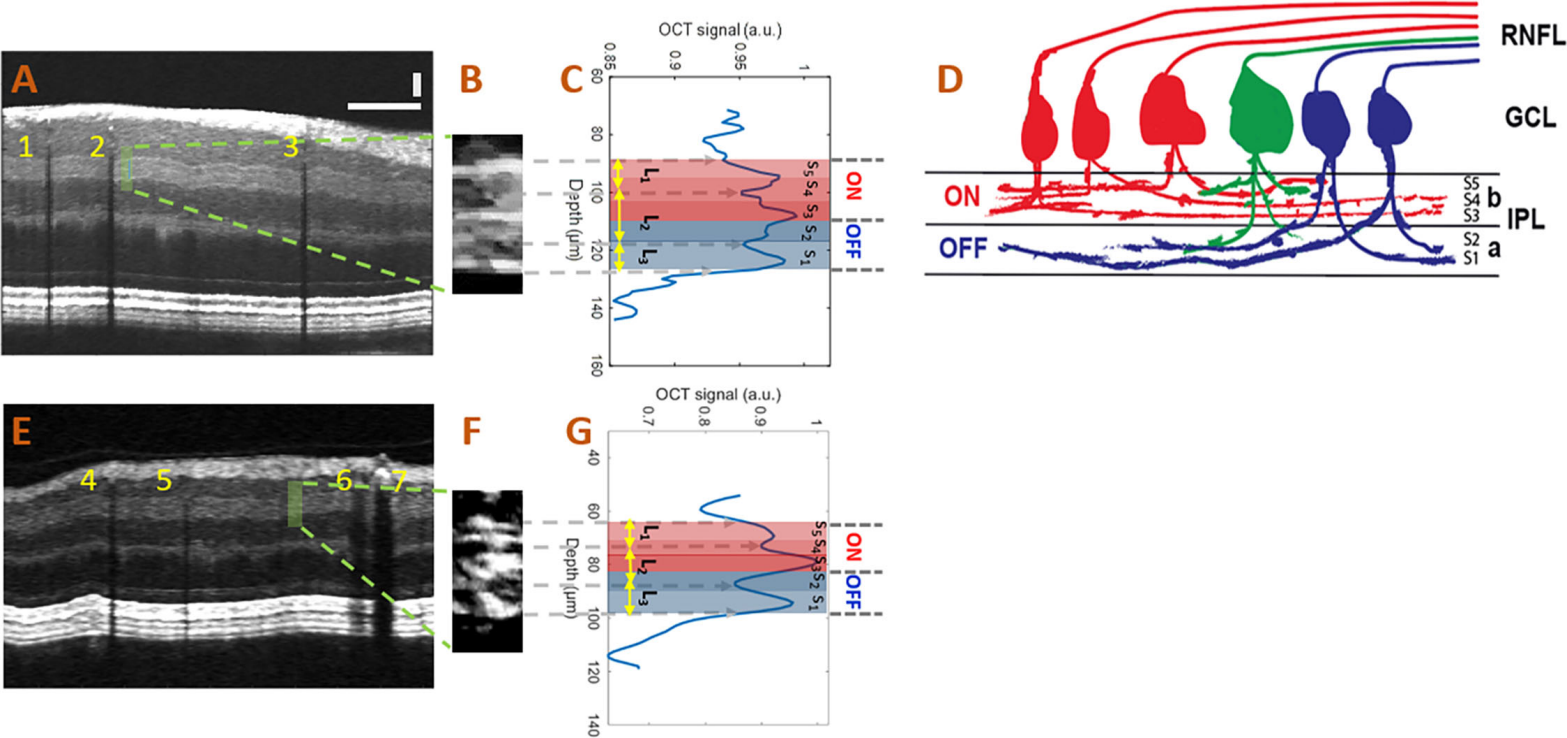
(**A**) A speckle-reduced vis-OCT image from a healthy eye. *Horizontal bar*: 500 µm; *vertical bar*: 50 µm. (**B**) Magnified view of the region highlighted by the *dashed box* in panel **A** (15 srA-lines segments). (**C**) Depth-resolved OCT amplitude profile of the IPL sublayers. We averaged 15 srA-lines, corresponding to approximately 88 µm along the lateral direction, within the highlighted region in panel **A**. (**D**) Illustration of the lamination of ganglion cells from RNFL to the IPL. The “red” ganglion cells (ON center) are laminating dendrites to the “b” sublamella of the IPL whereas “blue” cells (OFF center) laminate to the “a” sublamella. The “green” ganglion cell is bi-laminating. (**E**) A speckle-reduced vis-OCT image from a glaucoma eye. (**F**) A magnified view of the region highlighted by the *dashed box* in panel **E**. (**G**) Depth-resolved line profile of the glaucoma eye IPL sublayers.

Figure 2 illustrates the method of calculating IPL layer thickness and its underlying anatomical lamination. The initial reference data cube was acquired, and an srB-scan crossing the foveola was identified. Next, a quality index (QI)^28^ was computed for all srB-scans of the reference data cube that is superior to the srB-scan crossing the foveola, and a reference srB-scan (Fig. 2A) with the highest QI was selected to measure the IPL sublayer thicknesses. In the following imaging sessions, we identified srB-scan from the same location from the current data cube by pinpointing a blood vessel pattern specific to the reference sr-Bscan (e.g., vessels 1, 2, and 3 in Fig. 2A and vessels 4, 5, 6, and 7in Fig. 2E). All measurements were performed at the same distance from the selected vessel in the pattern in all scans of the subject. We then identified a segment of the IPL layer consisting of 15 srA-lines (Fig. 2B) to obtain a depth-resolved OCT amplitude (tissue reflectivity) profile (averaged A-line; a blue curve in Fig. 2C) to reduce noise in the actual thickness variation measurements. In the averaged A-line, three peaks and two valleys can be identified (Fig. 2C) corresponding to high- and low-intensity bands in the srB-scans (Figs. 2A and 2B). Such sublamination revealed in the srB-scan was well correlated with the anatomical five strata of the IPL reported in the literature.^8^^–^^12^ The hypothesized correspondence of the five strata (S1-S5) with measured IPL sublayers (L1-L3) was indicated in Figures 2C and 2D. A simplified sketch explaining the origin of the five strata as a stratification of dendrites from ON and OFF center ganglion cells and bi-laminating ganglion cells is shown in Figure 2D.

A representative segment of the IPL layers and corresponding tissue reflectivity profiles from all of the nine healthy subjects and five glaucoma patients are provided in Supplementary Figures S1 and S2, respectively. Table S1 summarizes the values of sublayers L1, L2, L3, and entire IPL thickness from Figures S1 and S2.

### Segmentation and Thickness Measurement of IPL Sublayers

With the improved vis-OCT imaging and post-data processing method, we can distinguish the sublayer structures in the IPL. Specifically, we can detect three hyper-reflective bands in the IPL, with the top and bottom bands setting the boundaries of the IPL. To measure variation in the fine lamella structure of the IPL, we identified three IPL sublayers that can be robustly measured from the averaged A-line profile with the following dividing lines (Fig. 2C): (1) sublayer L_1_ measured from the top IPL boundary to a minimum of the first valley from the top of IPL; (2) sublayer L_2_ measured between minima of the first and second from the top of IPL valleys; (3) sublayer L_3_ measured from the minimum of the second valley from the top of IPL to the bottom IPL boundary. Therefore L_1_ and L_3_ represent a part of the ON and OFF sublamina, respectively, whereas L_2_ includes both ON and OFF sublaminae. We measured the thicknesses of all the IPL sublayers manually from the averaged A-line profiles as number of pixels. The boundaries of the RNFL layer were also segmented manually at the same sampling locations as the IPL sublayer measurements by a single observer. Segmentation was performed using srB-scan for vis-OCT and regular B-scan for Cirrus. The number of pixels was multiplied by pixel height in each imaging mode (vis-OCT or Cirrus) to calculate the physical tissue thickness. The physical pixel height for both vis-OCT (1.08 µm/pixel) and Cirrus (1.95 µm/pixel) was provided by Opticent and Zeiss, respectively. The refractive index of n = 1.35 was used to measure distance in the retina.

### Statistical Analysis

Summary statistics were provided as median and twenty-fifth (Q1) and seventy-fifth (Q3) percentiles of the data for Table 1, median and the range for Tables 2 and 3, and mean and standard deviation (SD) for Tables 4 and 5. The Wilcoxon rank-sum test was used to compare the demographics between healthy and glaucomatous subjects. The coefficients of variation (CVs) of all three sublayers measured by vis-OCT and the entire IPL thickness were calculated to assess the intrasession and intersession repeatability. A linear mixed-effects model with a random intercept to account for intrasubject correlation was used to test whether parameters were different for glaucomatous and healthy subjects after adjusting for age. Outcome measures included both the entire IPL thickness and its individual three sublayers. Statistical analysis was performed using R software version 3.5.2. A *P* value <0.05 was considered statistically significant.

**Table 1.** Subject Demographics

Subjects	Number	Age (y), Median (Q1, Q3)	MD Range (dB), Median (Q1, Q3)	Global RNFL Thickness, Median (Q1, Q3)
Healthy	9	47.0 (33.0, 56.0)	0.23 (−0.1,0.7)	94.0 (89.0,105.0)
Glaucoma	5	64.0 (49.5,67.5)	−19.4 (−23.2, −11.0)	55.0 (52.5,69.5)
*P* value	—	0.02	0.002	0.004

**Table 2.** Intrasession Repeatability for Healthy and Glaucoma Subjects

	Entire IPL CV (%)		Sublayer L_1_ CV (%)		Sublayer L_2_ CV (%)		Sublayer L_3_ CV (%)	
	Median	Range	Median	Range	Median	Range	Median	Range
Healthy	3.1	0.0–4.3	5.6	5.1–6.2	6.9	3.5–8.8	5.6	5.4–5.6
Glaucoma	1.8	1.7–3.0	6.0	5.1–6.0	7.7	3.7–8.3	6.2	5.6–10.0
*P* value	0.44		0.95		0.69		0.007	

**Table 3.** Intersession Repeatability for Healthy Subjects

	Entire IPL CV (%)		Sub layer L_1_ CV (%)		Sublayer L_2_ CV (%)		Sublayer L_3_ CV (%)	
	Median	Range	Median	Range	Median	Range	Median	Range
Healthy	2.9	1.6–4.3	5.4	5.2–5.5	6.8	5.1–7.8	5.6	5.1–6.2

**Table 4.** Measured IPL Thickness and Calculated *P* Values Based on *Mixed-Effects Models* Comparing the Difference Between Glaucoma and Healthy Subjects

	Entire IPL Thickness (mm), Mean ± SD	Sublayer L_1_ Thickness (mm), Mean ± SD	Sublayer L_2_ Thickness (mm), Mean ± SD	Sublayer L_3_ Thickness (mm), Mean ± SD
Healthy	40.1 ± 1.7	11.4 ± 0.9	17.5 ± 1.4	11.3 ± 0.8
Glaucoma	36.2 ± 1.5	11.0 ± 0.9	14.2 ± 1.8	10.5 ± 0.8
*P* values	0.003	0.9	0.003	0.05

**Table 5.** Comparison of the Local RNFL Thickness at IPL Sampling Locations Between Commercial NIR-OCT and Experimental vis-OCT

	RNFL Thickness vis-OCT (µm), Mean ± SD	RNFL Thickness NIR-OCT (µm), Mean ± SD	Total Retina Thickness vis-OCT (µm), Mean ± SD	Total Retina Thickness NIR-OCT (µm), Mean ± SD
Healthy	24.5 ± 6.0	24.5 ± 6.2	323.6 ± 10	323.5 ± 9.7
Glaucoma	23.5 ± 3.9	23.4 ± 3.7	290.1 ± 17.7	290.6 ± 17.3
*P* values	0.94		0.99	

## Results

Subject demographics are summarized in Table 1. Glaucoma subjects were older and showed lower MD than healthy subjects. The global RNFL thickness was measured using a commercial NIR-OCT system.

All measurements expressed in Tables 2 through 4 were performed using the vis-OCT system. IPL sublayers were visible in all scans, both in glaucoma and healthy subjects. The imaging quality for healthy eyes was comparable to the glaucoma eyes without reaching statistical significance (*P* = 0.07): an average QI for srB-scans used in the IPL layers thickness calculations was 65.4 ± 1.0 for healthy and 65.0 ± 0.8 glaucoma subjects.

### Intrasession Repeatability

Intrasession repeatability results are summarized in Table 2. CVs showed good repeatability on all measured sublayers for both healthy and glaucomatous eyes. The variability of the entire IPL thickness measurements is significantly lower than the variability of the sublayer measurements for both healthy and glaucoma subjects. Among the sublayers, there was no significant difference in intrasession repeatability between glaucoma and healthy subjects.

### Intersession Repeatability

Intersession repeatability results are summarized in Table 3. CVs showed good repeatability on the entire IPL and the thickness of measured IPL sublayers in all healthy eyes. The values were similar to intrasession study values.

## IPL Sublayer Thickness

IPL sublayer thickness results are summarized in Table 4. The entire IPL was significantly thinner in glaucomatous eyes than healthy eyes (*P* = 0.003). After adjusting for the age difference using the mixed-effects model, the IPL sublayer L_2_ showed a statistically significant difference between glaucoma and healthy subjects, the sublayer L_3_ showed a marginal difference, and the sublayer L_1_ did not show a statistically significant difference. Age was not significant in the mixed effect models.

### Vis-OCT Axial Depth Calibration

To evaluate vis-OCT calibration, we measured RNFL thickness in the same sampling locations using commercial NIR-OCT and vis-OCT. The results shown in Table 5 indicate no significant difference between NIR-OCT and vis-OCT RNFL or total retinal thickness measurements.

## Discussion

The integrative properties of RGC dendritic structure and function are critical for visual function.^9^ In prior studies using NIR-OCT, the response of the IPL to glaucoma is considered insignificant compared with RNFL and combined GCL+IPL layers.^4^^,^^5^ In those studies, functional segregation of the ON and OFF pathways could not be investigated because of limited contrast and axial resolution. The newly developed vis-OCT^20^^,^^23^ and high-resolution NIR-OCT^18^^,^^19^ have demonstrated the capability of revealing IPL lamination, which enables quantitative analysis of IPL sublayers in vivo.

With our prototype vis-OCT system combined with the speckle reduction scanning technique, we demonstrated that the highly repeatable IPL sublayer quantification was possible. We considered three ways of measuring the layers. First, peak-to-peak distance, second valley-to-valley distance, and third peaks width measurements. The third method was rejected because the peak width measurements have been significantly affected by the thresholding of the peak borders (where the peak starts and ends in the middle of the slopes). The measurement of valleys-to-valleys was selected over peaks-to-peaks one after comparison between two methods and finding the more reliable (less variation) measurements were provided by the valley-to-valley method.

The median CV for the entire IPL is the lowest because the contrasts of the inner and outer borders of IPL are higher and more consistent than the intensity valleys we used to define the L_1_-L_2_ and L_2_-L_3_ borders. Nonetheless, all the median CVs (both intrasession and intersession assessments) are less than 8%, which implies that all the IPL sublayer measurements are highly repeatable for both healthy and glaucomatous eyes.

L_1_ (belongs to ON sublamella) did not show any significant difference between healthy and glaucoma. Both L_2_ (belongs to both ON and OFF sublamellae) and L_3_ (belongs to OFF sublamella) were statistically significantly thinner in eyes with glaucoma compared to healthy eyes. However, the detected difference in L_3_ thickness was 0.8um, which is well within the physical axial resolution range. This suggests that L_2_ plays a major role in IPL thinning with glaucoma. Also, our results imply that the OFF sublamellae play a larger role in IPL thinning with glaucoma.

The major limitation of the study was the small number of samples. We had only nine healthy eyes and five glaucomatous eyes. Also, all the eyes with glaucoma had advanced glaucomatous damage. Further investigation with a larger number of samples with a wider range of glaucomatous damage is warranted.

Another limitation was the use of intensity valleys as the borders between sublayers. Because there is a bright-dark-bright-dark-bright pattern, ideally three of the bright intensity peaks are segmented. However, this type of segmentation may introduce variability depending on how the thresholds along the slopes of the peaks are decided as the borders. To avoid such potential influence, we decided to use the intensity valleys as the borders between sublayers. Further investigation with a more advanced segmentation method is warranted.

Finally, sampling location variability can be a concern for assessing the longitudinal changes. With this pilot study, we did not register scans to minimize the sampling location variability for the intersession repeatability assessment. Therefore there is no guarantee that the sampling was done from nearly identical locations on separate sessions. The uncertainty in the OCT imaging location caused by eye motion in the range of 80 µm to 130 µm can be estimated from the data on the dispersion of the eye angle movement^29^^–^^31^ using an effective focal length of the human eye as 16.7 mm.^32^^,^^33^ Nonetheless, we observed high repeatability of the measurements, most likely because of the homogeneity of the sublamellae morphology of IPL.

In conclusion, this pilot study showed that speckle-reduction vis-OCT could provide a repeatable quantitative assessment of the IPL laminations noninvasively in a small cohort of healthy and glaucomatous eyes. We visualized the five reported morphological IPL strata from vis-OCT imaged IPL sublayers and found that the sublayer L_2_ played a major role in the IPL thinning in eyes with advanced glaucoma.

## Supplementary Material

Supplement 1
