# Supplementary material for: In Vivo Sublayer Analysis of Human Retinal Inner Plexiform Layer Obtained by Visible-Light Optical Coherence Tomography
Source: Invest Ophthalmol Vis Sci. 2022 Jan 13;63(1):18. doi: 10.1167/iovs.63.1.18 (PMC8762683; doi:10.1167/iovs.63.1.18)
Supplement: Supplement 1 [file iovs-63-1-18_s001.pdf]

Supplementary Material

In Vivo Sublayer Analysis Of Human Retinal Inner Plexiform Layer Obtained By Visible-Light Optical Coherence Tomography

Zeinab Ghassabi*[1], Roman V. Kuranov*[2,3], Joel S. Schuman[1,4,5], Ronald Zambrano[1], Mengfei Wu[1], Mengling Liu[1], Behnam Tayebi[1,5], Yuanbo Wang[3], Ian Rubinoff[2], Xiaorong Liu[6], Gadi Wollstein[1,4], Hao F. Zhang[2], and Hiroshi Ishikawa[1,4,7]

[1] Department of Ophthalmology, NYU Langone Health, New York, NY, United States.
[2] Department of Biomedical Engineering, Northwestern University, Evanston, IL, United States.
[3] Opticent Inc., Evanston, IL, United States.
[4] Department of Biomedical Engineering, New York University Tandon School of Engineering, Brooklyn, NY, USA
[5] Neuroscience Institute, NYU Langone Health, NY, NY, United States.
[6] Department of Biology, University of Virginia, Charlottesville, VA, United States
New York, NY, United States
[7] Department of Ophthalmology, Casey Eye Institute, Oregon Health & Science University, Portland, OR, United States

Funding: NIH: R01-EY013178, R01EY029121, R01EY026078, R44EY026466, Unrestricted grant from Research to Prevent Blindness

* These authors contributed equally to this work

Correspondence author: Dr. Hiroshi Ishikawa, ishikawh@ohsu.edu

H. F. Zhang, R. V. Kuranov, Y. Wang, and J. S. Schuman have financial interests in Opticent Inc.

Supplementary Figures

Figure S1. Representative segments of the IPL layer consisted of 15 srA-lines and corresponded depth-resolved OCT amplitudes of nine healthy.

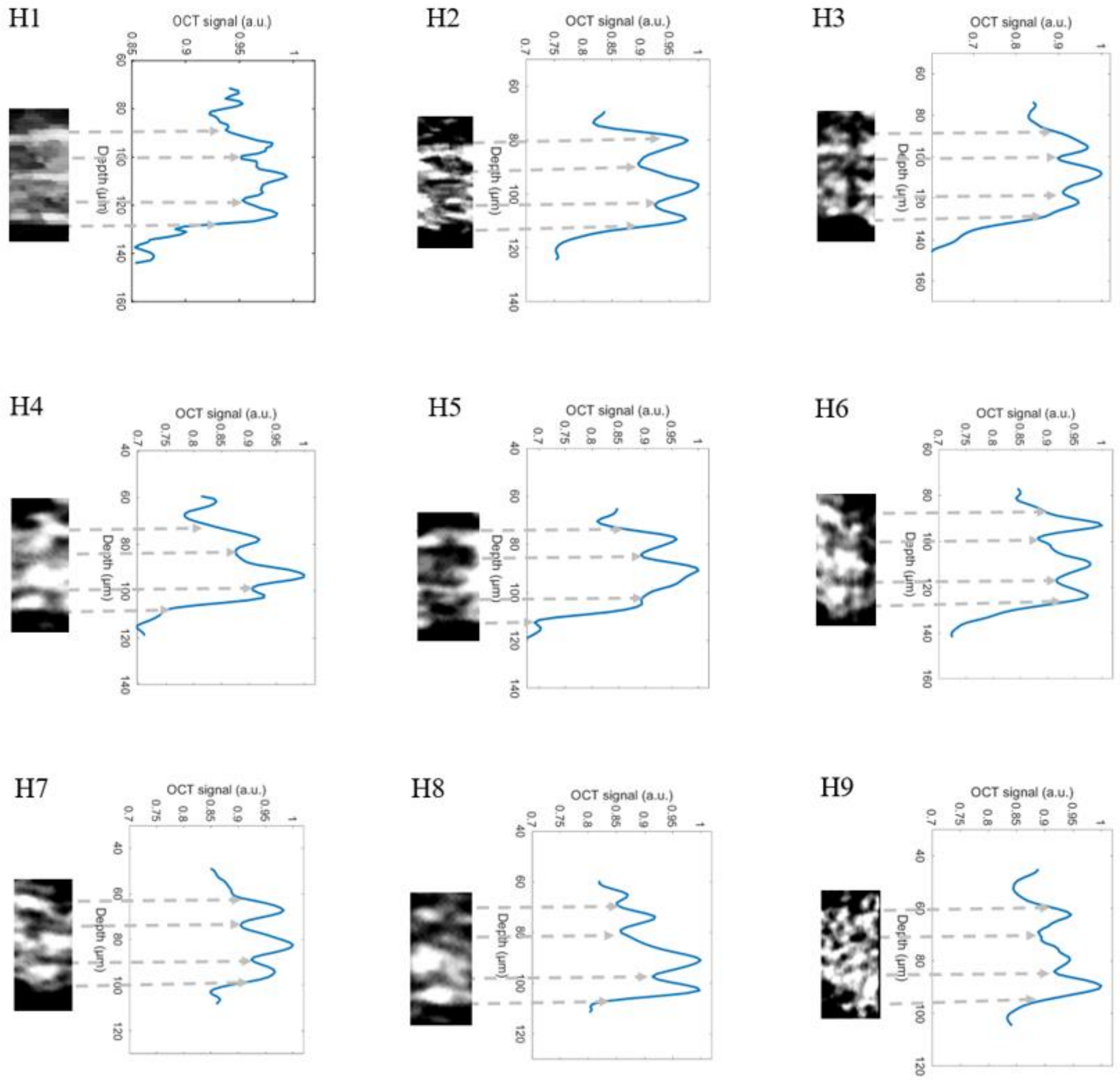

Figure S2. Representative segments of the IPL layer consisted of 15 srA-lines and corresponded depth-resolved OCT amplitudes of five glaucoma eyes.

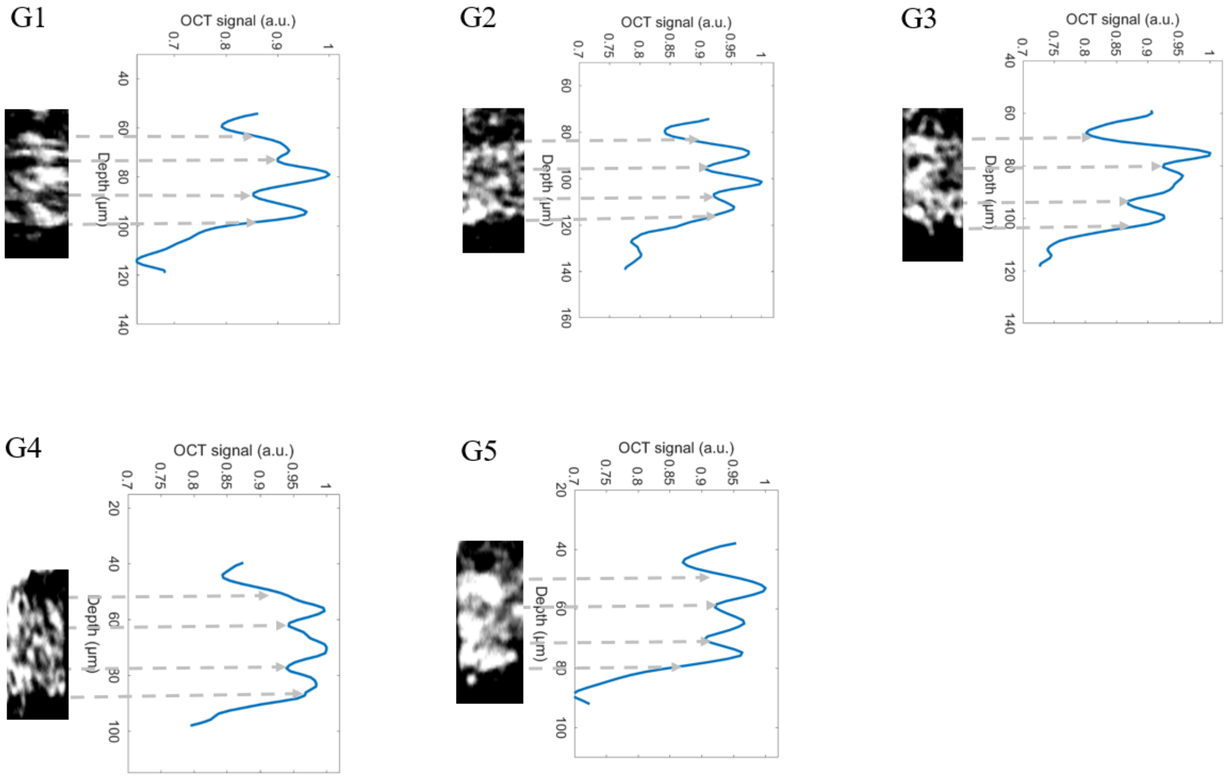

Supplementary Table

Table S1. Thickness measurements for sublayers L_1, L_2, L_3 and entire IPL of 9 healthy and 5 glaucoma subjects from the Figure S1 and S2 depth-resolved OCT amplitudes respectively.

Table S1. IPL sublayers and entire IPL measurements

Subject	Sublayer L1 (μm)	Sublayer L2 (μm)	Sublayer L3 (μm)	Entire IPL (μm)
**Healthy 1**	10.8	21.6	10.8	43.2
**Healthy 2**	11.9	16.2	10.8	38.9
**Healthy 3**	10.8	17.3	11.9	40.0
**Healthy 4**	10.8	16.2	10.8	37.8
**Healthy 5**	11.9	19.5	10.8	42.2
**Healthy 6**	11.9	19.5	10.8	42.2
**Healthy 7**	10.8	16.2	10.8	37.8
**Healthy 8**	10.8	19.5	10.8	41.1
**Healthy 9**	11.9	16.2	11.9	40.0
**Glaucoma 1**	10.8	14.1	11.9	36.8
**Glaucoma 2**	11.9	13.0	9.7	34.6
**Glaucoma 3**	10.8	14.1	10.8	35.7
**Glaucoma 4**	9.7	16.2	10.8	36.7
**Glaucoma 5**	10.8	14.1	9.7	34.6
